# Exercise training affects hemodynamics and exercise capacity in cases of heart failure with preserved ejection fraction: a non-randomized controlled trial in individuals aged 65–80 years

**DOI:** 10.3389/fcvm.2023.1246739

**Published:** 2023-10-30

**Authors:** Yousuke Sugita, Katsuhiko Ito, Yui Yoshioka, Ayano Kudo, Sota Arakawa, Satoshi Sakai

**Affiliations:** ^1^ Faculty of Health Sciences, Tsukuba University of Technology, Tsukuba, Japan; ^2^ Department of Rehabilitation, National Hospital Organization Matsumoto National Hospital, Matsumoto, Japan; ^3^ Department of Rehabilitation, Musashino General Hospital, Kawagoe, Japan

**Keywords:** heart failure with preserved ejection fraction, exercise training, peak oxygen uptake, peak stroke volume, peak heart rate, peak arteriovenous oxygen difference, ventilatory equivalent vs. carbon dioxide output slope, echocardiography

## Abstract

**Introduction:**

Exercise training is an established intervention method for improving exercise capacity and survival rates in patients with heart failure with preserved ejection fraction (HFpEF). However, most reports have focused on European and American patients, with limited data regarding the effects of exercise training on cardiac function, hemodynamics, and exercise capacity in East Asian patients. This study investigated the effects of exercise training on cardiac function, hemodynamics, and exercise capacity in Japanese patients aged 65–80 years with HFpEF.

**Methods:**

This single-center, open-label, non-randomized, controlled trial prospectively enrolled 99 outpatients. Eligibility criteria for HFpEF patients were an HFA score ≥5 in addition to clinical symptoms of heart failure and left ventricular diastolic dysfunction. Exercise training in the intervention group consisted of aerobic exercise and strength training thrice weekly for 5 months. Patients in the control group continued the usual treatment for 5 months. Resting cardiac function was evaluated using echocardiography. Peak oxygen uptake (peakVO_2_), ventilatory equivalent (VE) vs. carbon dioxide output (VCO_2_) slope, peak cardiac output index, and arteriovenous oxygen difference were calculated using cardiopulmonary exercise testing combined with impedance cardiography.

**Results:**

After 5 months of exercise training, remarkable interactions were observed, with peakVO_2_ as the primary outcome. Additionally, significant interactions were observed between hemodynamic indices and some echocardiographic parameters. The mean percentage change in peakVO_2_ from baseline was 8.3% in the intervention group. Fifteen study participants (30.1%) in the intervention group achieved a clinically meaningful change of 3.0 ml/min/kg (10% improvement) in peakVO_2_ from baseline. The group with 3.0 ml/min/kg or 10% improvement in peakVO_2_ from baseline had a considerably lower prevalence of diabetes mellitus and VE vs. VCO_2_ slope and considerably higher left atrial-global longitudinal strain values than the group without any notable improvements.

**Conclusions:**

Although exercise training can help improve exercise intolerance in Japanese patients aged 65–80 years with HFpEF, its benefits are limited. Our results suggest that HFpEF, complicated by diabetes mellitus and decreased ventilatory efficiency during exercise, may require reconsideration of intervention strategies. This trial was registered with the University Hospital Medical Information Network, a trial registry in Japan (registration number: UMIN000045474).

## 1. Introduction

The prevalence of heart failure (HF) has been increasing annually worldwide (1). HF with preserved ejection fraction (HFpEF) accounts for approximately 50% of HF cases (2). Deterioration of the left ventricular (LV) structure and exercise intolerance are clinical symptoms of HFpEF and closely associated with mortality and hospital readmission rates (3–5). Thus, there are clinical implications for the establishment of methods to improve the LV structure, such as LV hypertrophy (LVH), LV enlargement, and exercise intolerance in patients with HFpEF.

Exercise training is an established intervention method for improving the objectively measured peak oxygen uptake (peakVO_2_) and quality of life in patients with HFpEF (6–8). Meta-analyses based on multiple randomized controlled trials have shown that exercise training interventions improve peakVO_2_ and some hemodynamic responses [e.g., heart rate (HR) response and blood pressure] in patients with HFpEF (9). However, most reports are from Western populations and lack data on the effects of exercise training interventions on cardiac function, hemodynamic response during exercise, and exercise capacity in East Asian patients with HFpEF. In a registry study on HFpEF conducted in Japan, the median age of HFpEF patients was 80 years old, and the mean body mass index (BMI) was 23.9 ± 4.7 kg/m^2^, indicating a distinctly different cardiovascular phenotype from the data of Westerners (10, 11). To the best of our knowledge, the study by Fu et al. is the only report on East Asian patients with HFpEF (12). In this study, the mean age of the patients was 60.5 years, which may be younger than that of the general population of patients with HFpEF. In addition, peakVO_2_ at baseline had a relatively maintained exercise capacity with a mean of approximately 15 ml/min/kg, and no data were presented on comorbidities that may affect exercise training efficacy, such as increased brain natriuretic peptide (BNP) levels, the prevalence of sarcopenia, anemia, atrial fibrillation (AF), and chronotropic incompetence. HFpEF results in multiple-organ failure and has many phenotypes. The consideration of these comorbidities is an important component of treatment (13), and further investigation is needed to determine the effect of exercise training on East Asian patients with HFpEF, including older individuals and those with comorbidities.

Therefore, we hypothesized that an exercise training intervention may improve LV structure and function, hemodynamics, and exercise capacity. We aimed to investigate this hypothesis in older Japanese patients with HFpEF and various comorbidities.

## 2. Materials and methods

### 2.1. Study design and participants

This was a prospective, single-center, non-randomized study. The details of the study protocol, diagnostic criteria for HFpEF (14, 15), and sample size calculations (16) are provided in the Supplementary Material. The primary outcome was a change in peakVO_2_ after 5 months, with the minimal clinically significant difference set at 3.0 ml/kg/min. Secondary outcomes were changes in the ventilatory equivalent (VE) vs. carbon dioxide output (VCO_2)_ slope, LV diastolic function indices, and BNP after 5 months.

Written informed consent was obtained from all the participants. This study was conducted according to the guidelines of the Declaration of Helsinki. The study protocol was approved by the Institutional Review Board of Tsukuba University of Technology, Tsukuba City, Japan (approval number: 202108).

### 2.2. Exercise training program and usual care

Exercise training was performed three times per week for 5 months. The exercise program consisted of approximately 5 min of warm-up, 30 min of aerobic exercise, 20 min of resistance training, and 5 min of cool-down (17). Details of aerobic exercise and resistance training are provided in the Supplementary Material. The control group continued the usual care for 5 months. Patients who required an increase in the dose of oral medications, such as diuretics, during the usual care period were excluded from the analysis.

### 2.3. Anthropometric parameters, biochemical analysis, and blood pressure

BMI and body surface area (BSA) were calculated by measuring height and weight (Supplementary Material). Overweightness and obesity were determined from the calculated BMI based on the criteria of the World Health Organization (WHO) for obesity (18). BSA was calculated using the formula described by Du Bois et al. (Supplementary Material) (19).

Blood was drawn from the study participants after 12 h of fasting and before ingesting medications. After collecting 10 ml of blood, their BNP, triglycerides, total cholesterol, high-density lipoprotein cholesterol, low-density lipoprotein cholesterol, hemoglobin A1c, hemoglobin, fasting plasma glucose, plasma glucose, and insulin levels were measured (Supplementary Material).

We also performed a homeostasis model assessment of insulin resistance (20) and determined the estimated glomerular filtration rate (21) (Supplementary Material). Anemia was defined as a hemoglobin level of <13 g/dl in men and <12 g/dl in women (WHO criteria) (22).

Systolic and diastolic blood pressures were measured in the arms of the seated participants after a 20-min rest using an automatic blood pressure monitor (HEM-7220, Omron Healthcare Co., Ltd., Kyoto, Japan). Hypertension, diabetes mellitus, and dyslipidemia were diagnosed according to the Japanese diagnostic criteria (Supplementary Material) (23).

### 2.4. Echocardiography

Structural and functional abnormalities of the LV and left atrium (LA) were assessed using echocardiography (ACUSON SC2000; 4V1c and 4Z1c probes; Siemens Japan K.K., Tokyo, Japan) with individuals in the left decubitus position. The LV posterior wall thickness at end-diastole, interventricular septal thickness at end-diastole, LV end-diastolic diameter, LV end-systolic diameter, LV diameter, and LV wall thickness were recorded in M-mode. The LV end-diastolic and end-systolic volumes were measured using the biplane-modified Simpson method. Relative wall thickness (RWT) and LV myocardial weight were calculated using Devereux's formula (24). The formulas for calculating LV ejection fraction (LVEF) and stroke volume (SV) are shown in the Supplementary Material.

For epicardial adipose tissue thickness measurements, all participants underwent echocardiography, as proposed by Iacobellis et al. (Supplementary Material) (25).

The LV inflow parameters were obtained using pulse-wave tissue Doppler in the apical four-chamber view. Peak early flow velocity, late diastolic flow velocity, the ratio of peak early velocity to late diastolic flow velocity, and early diastolic flow wave deceleration time were assessed. Pulsed-wave tissue Doppler imaging was performed to obtain the peak early diastolic tissue velocity in the septal and lateral aspects of the mitral annulus. The mitral inflow early diastolic velocity ratio to the average velocity from the septal and lateral sides of the mitral annulus was calculated to estimate the LV filling pressure.

Pulmonary artery systolic pressure was estimated according to the methods presented in Supplementary Material (26). A detailed evaluation of mitral regurgitation and its severity is also included (27, 28).

Based on a report by Lang et al. (29), LVH was defined as an LV mass index of >115 g/m^2^ for men and >95 g/m^2^ for women. LV concentric remodeling was defined as LVH (−) and RWT > 0.42, LV eccentric hypertrophy was defined as LVH (+) and RWT < 0.42, and LV concentric hypertrophy was defined as LVH (+) and RWT > 0.42.

The LA volume (LAV) was measured in three different sequences of the cardiac cycle. The maximum LAV was measured immediately before the mitral valve opened, the pre-A LAV (before atrial contraction) was determined at the onset of atrial contraction (*P*-wave peak electrocardiogram), and the minimum LAV was measured when the mitral valve was closed. All volumes were determined according to the biplane method in the four- and two-chamber views. The LA emptying fraction, which is the comprehensive reservoir function of LA, was calculated using the formula shown in the Supplementary Material. The LAV index was also calculated using the methods and formulas presented in Supplementary Material (30).

### 2.5. Speckle-tracking imaging

LV myocardial deformation was assessed using a two-dimensional speckle-tracking technique in three apical views at a temporal resolution of 60–90 frames/s (Supplementary Material). The LV global longitudinal strain (LV-GLS) represented LV shortening in the longitudinal plane (31). Furthermore, LA speckle-tracking imaging, longitudinal strain, and strain rate curves were generated for each of the six atrial segments obtained from the apical four- and two-chamber views. The peak LA-global longitudinal strain (LA-GLS) value was calculated by averaging the values observed in all six LA segments analyzed (32).

### 2.6. Measurement of exercise capacity and hemodynamic response

Exercise capacity was measured through cardiopulmonary exercise testing (CPET) with a symptomatic limit using an ergometer (232C-XL; Combi Co., Ltd., Tokyo, Japan). The peakVO_2_ (33), work rate at peak exercise (peak watt), anaerobic threshold (ATVO_2_) (34), and work rate at exercise (AT watt) were measured according to the methods described in the Supplementary Material. The VE vs. VCO_2_ slope was measured by selecting the range from the point at which VE began to increase during ramp loading to the point of respiratory compensation. The heart rate recovery (HRR) and oxygen pulse were calculated using the methods presented in the Supplementary Material.

The hemodynamic response from sitting to peak exercise was measured using a non-invasive transthoracic bioimpedance device (PhysioFlow PF-05 Lab1; Manatec Biomedical, Paris, France) during CPET. Impedance cardiography using PhysioFlow has been reported to be a noninvasive cardiac output assessment method that is highly correlated with the direct Fick method (35). The measurement parameters in PhysioFlow were SV and HR. The SV index (SI), cardiac output index (CI), and arteriovenous oxygen difference (a-vO_2_ diff) were calculated using the methods described in the Supplementary Material. The methods used to determine chronotropic incompetence and abnormal HRR values are also described in the Supplementary Material (36, 37).

### 2.7. Measurement of physical activity and nutrition intake

Daily physical activity was estimated from the magnitude and frequency of the acceleration signal detected at 32 Hz using a pedometer with multiple memory accelerometers (Lifecorder; Suzuken Co., Ltd., Nagoya, Japan). We assumed that step count values >20,000 steps/day and <500 steps/day were not routine (38). Carbohydrates, fats, and proteins were assessed for the daily total energy intake (Supplementary Material) (39).

### 2.8. Diagnosis of sarcopenia

Sarcopenia was defined according to the Asian Working Group for Sarcopenia 2019 (40): a skeletal muscle mass index (SMI) of <7.0 kg/m^2^ for men and <5.7 kg/m^2^ for women, a grip strength of <28 kg for men and <18 kg for women, or a five-times chair-stand test (SS-5) time ≥12 s. The SMI, grip strength, and SS-5 were measured as described in the Supplementary Material.

### 2.9. Adverse events

Adverse events associated with interventions for exercise training include acute coronary syndrome, cardiac arrest, chest symptoms (e.g., chest pain, shortness of breath, and palpitations), hypoglycemia, arrhythmia, dizziness, headache, severe fatigue with a Borg scale rating >15, and skeletal muscle pain. These items were drafted based on a study by Kitzman et al. (41), with some items being expanded independently.

### 2.10. Statistical analysis

Normally distributed data are expressed as the mean ± standard deviation, whereas nominal data are expressed as percentages. SPSS version 27 (IBM Japan, Ltd., Tokyo, Japan) was used for all statistical analyses. Using a two-tailed test, the significance level was set at *p* < 0.05. An unpaired Student's t-test, *χ*^2^ test, and Fisher's exact test were used to compare the differences in baseline data between the two groups. The intervention group was further classified into two subgroups with or without a clinically meaningful change of 3.0 ml/min/kg (16) or a 10% improvement in peakVO_2_ (42) from baseline; clinical characteristics in these subgroups were compared using an unpaired Student's t-test and *χ*^2^ test. The presence or absence of diabetes mellitus that was found to have a significant difference in this analysis was used as a covariate in the repeated two-way analysis of variance. A repeated two-way analysis of variance was performed to examine whether the variability of each variable, such as the primary and secondary outcomes, differed between the two groups before and after the intervention. A simple main-effect test was performed using the Bonferroni method to determine if the group × time interaction was statistically significant.

## 3. Results

### 3.1. Study participants

**Figure 1:**
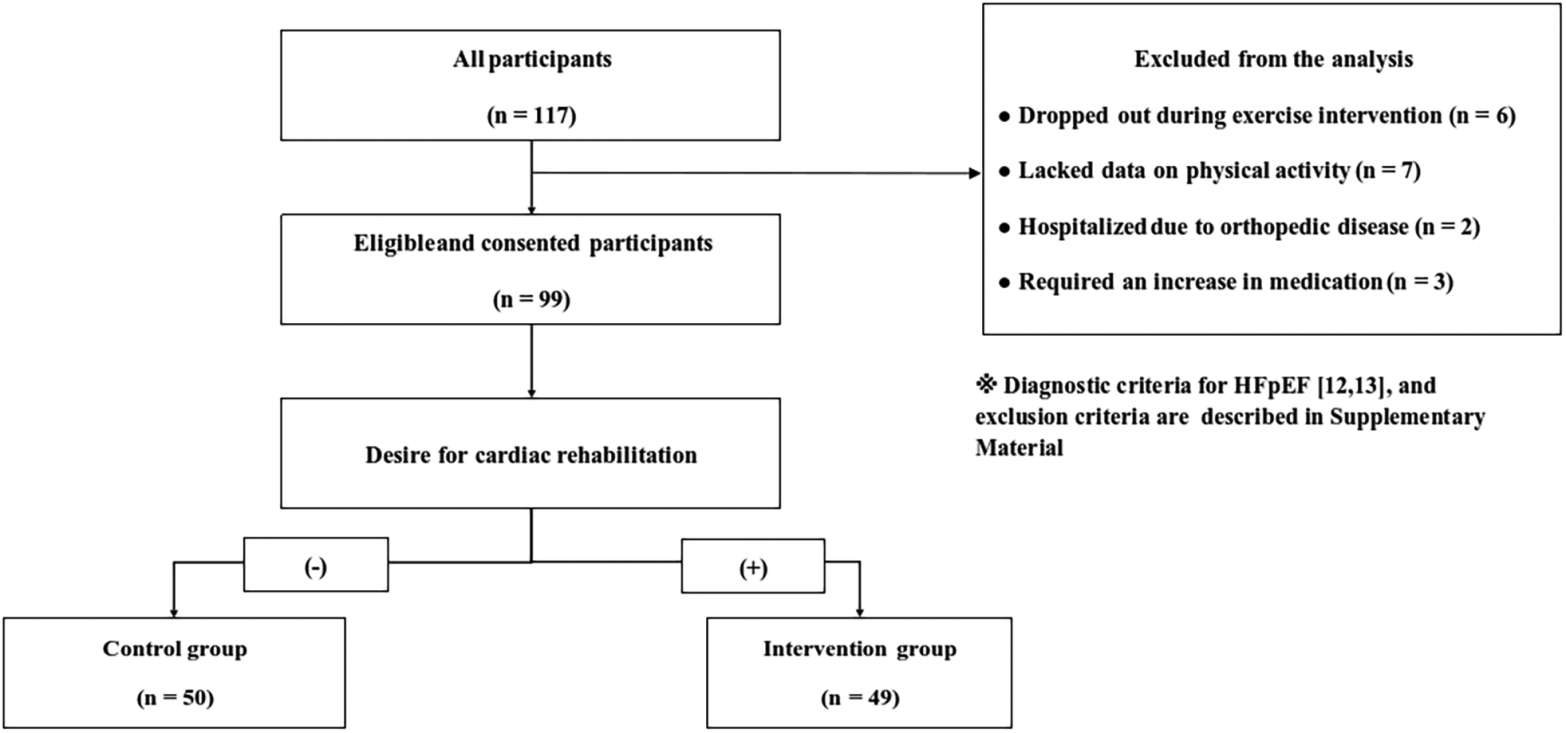
Study design and exclusion criteria. HFpEF, heart failure with preserved ejection.

Between 2016 and 2021, 117 individuals who did not meet the exclusion criteria were enrolled in the study, and 99 were included in the final analysis (Figure 1). Of the 117 registrants, 18 were excluded from the analysis (six dropped out during the exercise intervention, seven lacked data on physical activity, two were hospitalized due to orthopedic disease, and three required an increase in medication).

### 3.2. Comparison of all data in both groups at baseline

As seen in Table 1*,* at baseline, treatment with ezetimibe was markedly higher in the intervention group. Other data, such as echocardiography and CPET, showed no significant differences between the groups at baseline. The major comorbidities in the 99 patients with HFpEF were old myocardial infarction (26%), AF (58%), chronic obstructive pulmonary disease (15%), anemia (16%), hypertension (68%), dyslipidemia (54%), diabetes mellitus (52%), obesity (8%), overweightness (57%), and sarcopenia (16%).

**Table 1:** Characteristics of all participants.

Characteristics	Control group (*n* = 50)	Intervention group (*n* = 49)
HFA-PEFF score	5.4 ± 0.5	5.3 ± 0.5
NYHA functional classification		
Class Ⅱ	52	51
Class Ⅲ	48	49
Age (years)	73 ± 4	74 ± 4
Male (%)	48	49
Anthropometric parameters		
Weight (kg)	69 ± 8	69 ± 9
Body mass index (kg/m^2^)	26.2 ± 2.5	26.1 ± 2.6
Body surface area (m^2^)	1.73 ± 0.14	1.74 ± 0.16
Waist circumference (cm)	118 ± 9	119 ± 11
Obesity (%)	8	8
Overweight (%)	58	55
Energy intake		
Total energy intake (Kcal/day)	1,817 ± 229	1,821 ± 282
Carbohydrate intake (%)	60	59
Fat intake (%)	27	29
Protein intake (%)	13	12
Physical activity		
Steps (steps/days)	4,994 ± 616	5,158 ± 2,067
Movement related to calorie consumption (kcal/days)	197 ± 42	201 ± 79
Sarcopenia		
Skeletal muscle mass (kg/m^2^)	7.1 ± 0.9	6.9 ± 0.9
Hand grip (kg)	22.7 ± 4.8	23.4 ± 6
Sit to stand-5 (seconds)	9 ± 1	9 ± 1
Sarcopenia (%)	12	19.2
Preference and Medication		
Smoker (%)	26	24
Angiotensin-converting-enzyme inhibitor (%)	68	61
Angiotensin II Receptor Blocker (%)	56	49
*β* blocker (%)	62	63
Calcium-channel blocker (%)	50	45
Diuretic (%)	48	49
Statin (%)	90	92
Fibrate (%)	2	2
Ezetimibe (%)	38	80*
Biguanide (%)	40	41
Sulphonylurea (%)	38	33
*α*-glucosidase inhibitor (%)	12	8
Sodium glucose cotransporter-2 inhibitor (%)	12	14
Dipeptidyl peptidase-4 inhibitor (%)	10	12
Biochemical analysis and blood pressure		
Total Cholesterol (mg/dl)	223 ± 13	223 ± 12
Low-density lipoprotein cholesterol (mg/dl)	140 ± 13	143 ± 13
High-density lipoprotein cholesterol (mg/dl)	51 ± 7	50 ± 10
Triglyceride (mg/dl)	159 ± 19	153 ± 15
HbA1c (%)	7.5 ± 2.4	7.2 ± 2.3
Fasting plasma glucose (mg/dl)	131 ± 29	127 ± 34
HOMA-IR (%)	2.5 ± 1.1	2.4 ± 1.4
eGFR at cystatin C (ml/min/1.73 m^2^)	54 ± 8	55 ± 6
Brain natriuretic peptide (pg/ml)	189 ± 43	197 ± 40
Anemia (%)	12	20
Hypertension (%)	70	65
Dyslipidemia (%)	60	47
Diabetes mellitus (%)	52	51
Echocardiography		
Presence of concentric remodeling (%)	76	76
Presence of eccentric hypertrophy (%)	84	71
Presence of concentric hypertrophy (%)	60	47
Mitral regurgitation		
Mild (%)	22	24
Moderate (%)	28	27
Cardiopulmonary exercise testing		
Peak respiratory exchange ratio	1.13 ± 0.02	1.13 ± 0.02
PeakVO_2_ (ml/min/kg)	14.3 ± 4.3	13.8 ± 3.7
Peak work rate (watt)	79 ± 24	76 ± 21
AT-VO_2_ (ml/min/kg)	10.3 ± 2.7	9.6 ± 2.0
VE vs. VCO_2_ slope	36.7 ± 2.1	37.1 ± 2.1
Chronotropic incompetence (%)	28	27
Abnormality of HRR (%)	64	63
Reason for end of exercise load		
Leveling off of VO_2_	0	0
SBP decreased by 10 mmHg with exercise load and SBP was 250 mmHg or more	32	24
RPE_R_ and RPE_L_ > 17	12	29*
Pedal speed of<50 rpm	36	31
Request for termination from study participants	20	16

NYHA, New York Heart Association; eGFR, estimated glomerular filtration rate; HOMA-IR, homeostasis model assessment of insulin resistance; E/e′, ratio of the mitral inflow early diastolic velocity to the mean e′ velocity from the septal and lateral sides of the mitral annulus; peakVO_2_, peak oxygen uptake; AT, anaerobic threshold; VE vs. VCO_2_ slope, ventilatory equivalent vs. carbon dioxide output slope; HRR, heart rate recovery; SBP, systolic blood pressure; RPE_R_, Rating of perceived exertion on the respiratory; RPE_L_, Rating of perceived exertion on the lower extremity; HbA1c, glycated hemoglobin; HFA-PEFF, Heart Failure Association-Pre-test assessment, Echocardiography and natriuretic peptide, Functional testing, Final etiology.

Data are expressed as mean ± standard deviation, and nominal variables are expressed as percentages.

* *P* < 0.05 vs. Control group.

### 3.3. Changes in peakVO_2_ as a primary outcome

**Figure 2:**
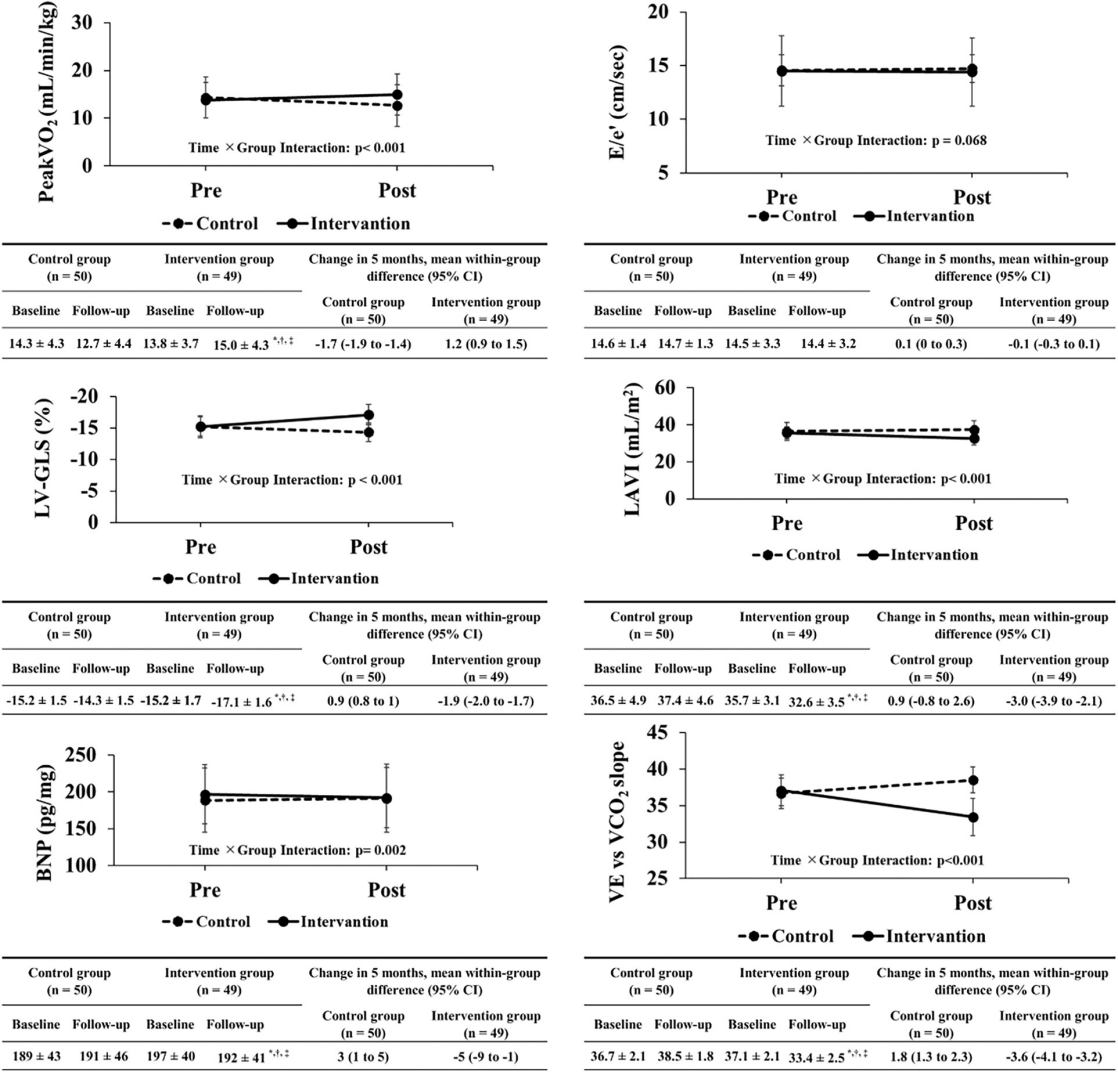
Changes in primary and secondary outcomes between both groups before and after exercise training intervention. peakVO_2_, peak oxygen uptake; E/e′, ratio of the mitral inflow early diastolic velocity to the mean e′ velocity from the septal and lateral sides of the mitral annulus; e′, peak early diastolic tissue velocity; LV-GLS, left ventricular global longitudinal strain; LAVI, left atrial volume index; BNP, brain natriuretic peptide; VE vs. VCO_2_ slope, ventilatory equivalent vs. carbon dioxide output slope.

**Figure 3:**
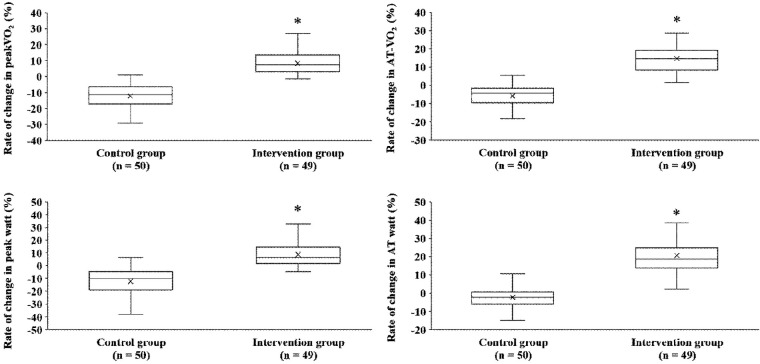
Comparison of percent changes in exercise performance between both groups before and after exercise training intervention. peakVO_2_, peak oxygen uptake; AT, anaerobic threshold.

After the exercise training intervention, remarkable observations were made in terms of peakVO_2_, an indicator of exercise capacity. A simple main-effect test of time and groups showed notable changes in the intervention group after the intervention (Figure 2). The mean percentage change in peakVO_2_ from baseline was 8.3% in the intervention group and −12.4% in the control group (Figure 3). Fifteen study participants (30.1% of the intervention group) achieved a clinically meaningful improvement of 10% in peakVO_2_ from baseline (Table 2). In contrast, none of the patients in the control group achieved such an improvement. The group with a 10% improvement in peakVO_2_ had a considerably lower prevalence of diabetes mellitus and a low VE vs. VCO_2_ slope but considerably higher LA-GLS values than the group without improvement (Table 2).

**Table 2:** Clinical characteristics of intervention groups with or without a clinically meaningful change in peakVO_2_ from baseline.

Characteristics	Non-improvement group (*n* = 34)	Improvement group (*n* = 15)
Age (years)	74 ± 4	75 ± 5
Male (%)	47	53
NYHA functional classification		
Class Ⅱ	50	53
Class Ⅲ	50	47
Comorbidities		
Old myocardial infarction (%)	29	20
Atrial fibrillation (%)	59	67
Chronic obstructive pulmonary disease (%)	15	13
Anemia (%)	29	13
Hypertension (%)	65	67
Dyslipidemia (%)	44	53
Diabetes mellitus (%)	71	7*
Obesity (%)	3	20
Overweight (%)	53	60
Sarcopenia (%)	15	7
Smoker (%)	26	20
Movement related to calorie consumption (kcal/days)	204 ± 78	194 ± 82
eGFR at cystatin C (ml/min/1.73 m^2^)	55 ± 6	55 ± 7
Brain natriuretic peptide (pg/ml)	191 ± 36	184 ± 45
Echocardiography data		
Left atrial ejection fraction (%)	45.2 ± 4.2	46.8 ± 4.2
Left ventricular global longitudinal strain (%)	−15.4 ± 1.8	−14.8 ± 1.5
Left atrial global longitudinal strain (%)	28.5 ± 3.5	32.5 ± 3.6*
Presence of concentric remodeling (%)	71	87
Presence of eccentric hypertrophy (%)	74	67
Presence of concentric hypertrophy (%)	44	53
Mitral regurgitation mild (%)	24	27
Moderate (%)	26	27
Estimated pulmonary artery systolic pressure (mmHg)	43.4 ± 10.5	41.5 ± 11.1
Peak oxygen uptake at Baseline (ml/min/kg)	13.5 ± 3.8	14.5 ± 3.5
Chronotropic incompetence (%)	29	20
Abnormality of HRR (%)	68	53
VE vs. VCO_2_ slope	37.7 ± 2.1	35.6 ± 1.2*

Data are expressed as mean ± standard deviation, and nominal variables are expressed as percentages.

* *P* < 0.05 vs. Non-improvement group.

NYHA, New York Heart Association; eGFR, estimated glomerular filtration rate; HRR, heart rate recovery; VE vs. VCO_2_ slope, ventilatory equivalent vs. carbon dioxide output slope.

### 3.4. Changes in secondary outcomes

After exercise training intervention, remarkable interactions were observed in the LV-GLS, left atrial volume index, BNP, and VE vs. VCO_2_ slope (Figure 2). These variables were also notable for time and groups in the simple main effects tests, showing extensive improvements in the intervention group (Figure 2). In contrast, no significant interaction was observed in E/e′ as an index of LV diastolic function before and after exercise training intervention.

### 3.5. Impact of exercise training intervention on echocardiography data

After the exercise training intervention, remarkable interactions were observed between the LA-GLS, RWT, and epicardial adipose tissue thickness (Table 3). These variables were also notable for time and groups in the simple main effects tests, showing a marked improvement in the intervention group. In contrast, no significant interactions were observed among LV mass index (LVMI), LVEF, left atrial emptying fraction, LV end-diastolic volume index (LVEDVI), and LV end-systolic volume index (LVESVI).

**Table 3:** Changes in echocardiography data between both groups before and after cardiac rehabilitation intervention.

	Control group (*n* = 50)		Intervention group (*n* = 49)		Change in 5 months, mean within-group difference (95% CI)	
Parameters	Baseline	Follow-up	Baseline	Follow-up	Control group (*n* = 50)	Intervention group (*n* = 49)
Epicardial adipose tissue thickness (mm)	8.4 ± 0.9	9.0 ± 1.0	8.4 ± 0.6	7.5 ± 0.6*^,^^†,‡^	0.6 (0.5 to 0.7)	−0.8 (−0.8 to −0.9)
Interventricular septal thickness at end diastole (mm)	10.1 ± 1.0	10.2 ± 1.0	10.0 ± 0.9	10.0 ± 0.9*	0.11 (0.09 to 0.14)	0.02 (−0.02 to 0.07)
Posterior wall thickness at end diastole (mm)	10.2 ± 1.1	10.3 ± 1.1	10.0 ± 0.9	10.0 ± 0.9*	0.1 (0.1 to 0.2)	−0.002 (−0.029 to 0.033)
Left ventricular end-diastolic diameter (mm)	46.1 ± 1.9	46.0 ± 1.8	45.9 ± 3.0	45.8 ± 2.8	−0.1 (−0.2 to 0.1)	−0.13 (−0.29 to 0.03)
Left ventricular end-systolic diameter (mm)	29.6 ± 2.3	29.8 ± 2.4	28.9 ± 3.4	28.9 ± 3.4*	0.2 (0.1 to 0.3)	0.02 (−0.02 to 0.06)
Left ventricular end-diastolic volume index (mL/m^2^)	56.7 ± 6.1	56.2 ± 6.1	56.5 ± 9.5	56.4 ± 8.4	−0.5 (−1.4 to 0.3)	−0.1 (−1.3 to 1.2)
Left ventricular end-systolic volume index (mL/m^2^)	19.8 ± 3.8	20.0 ± 3.9	18.7 ± 5.0	18.9 ± 5.3	0.3 (−1.4 to 0.3)	0.2 (−0.2 to 0.7)
Left ventricular ejection fraction (%)	65.0 ± 6.0	64.3 ± 6.2	66.5 ± 8.2	66.3 ± 8.3	−0.7 (−1.2 to −0.3)	−0.2 (−0.6 to 0.1)
Left atrial ejection fraction (%)	47.5 ± 6.1	47.2 ± 6.5	45.7 ± 4.2	46.9 ± 6.2	−0.3 (−3.0 to 2.4)	1.3 (−0.5 to 3.0)
SI (mL/m^2^)	36.9 ± 5.5	36.1 ± 5.4	37.8 ± 8.8	37.5 ± 7.8	−0.8 (−1.4 to −0.1)	−0.3 (−1.2 to 0.6)
CI (L/min/m^2^)	2.5 ± 0.4	2.4 ± 0.4	2.7 ± 0.7	2.5 ± 0.5	−0.1 (−0.2 to −0.1)	−0.2 (−0.3 to −0.2)
Left ventricular mass index (g/m^2^)	117 ± 16	118 ± 18	115 ± 20	115 ± 20	1.1 (−0.8 to 3.0)	0.6 (−1.9 to 3.1)
Relative wall thickness	0.44 ± 0.05	0.45 ± 0.05	0.44 ± 0.04	0.44 ± 0.04*	0.006 (0.003 to 0.008)	0.001 (−0.001 to 0.003)
E (cm/sec)	59.7 ± 9.1	58.0 ± 9.4	60.9 ± 10.8	62.4 ± 10.6*^,†,‡^	−1.7 (−2.3 to −1.2)	1.4 (1.2 to 1.7)
A (cm/sec)	77.0 ± 13.1	74.9 ± 13.3	76.9 ± 7.1	77.5 ± 6.9*^,†^	−2.1 (−2.7 to −1.5)	0.6 (0.5 to 0.8)
E/A	0.8 ± 0.1	0.8 ± 0.1	0.8 ± 0.1	0.8 ± 0.1*^,†^	−0.001 (−0.008 to 0.006)	0.012 (0.011 to 0.015)
DcT (cm/sec)	236 ± 24.8	248 ± 24	241 ± 26	237 ± 25*^,†,‡^	11.6 (10.1 to 13.0)	−4.2 (−5.4 to −3.1)
Lateral e′ (cm/sec)	5.4 ± 1.1	5.1 ± 1.1	5.5 ± 2.0	5.6 ± 2*^,†^	−0.3 (−0.3 to −0.2)	0.1 (0.1 to 0.2)
Medial e′ (cm/sec)	2.9 ± 1.0	2.9 ± 1.1	3.6 ± 1.5	3.7 ± 1.5*^,†,‡^	−0.1 (−0.1 to 0.03)	0.1 (0.05 to 0.1)
Mean e′ (cm/sec)	4.2 ± 1.0	4.0 ± 1.1	4.5 ± 1.7	4.7 ± 1.7*^,†,‡^	−0.2 (−0.2 to −0.1)	0.1 (0.1 to 0.2)
E/e′ (cm/sec)	14.6 ± 1.4	14.7 ± 1.3	14.5 ± 3.3	14.4 ± 3.2	0.1 (−0.03 to 0.3)	−0.1 (−0.3 to 0.1)
Peak tricuspid regurgitation velocity (m/s)	2.8 ± 0.3	3.0 ± 0.3	2.8 ± 0.5	2.8 ± 0.5*^,†,‡^	0.1 (0.1 to 0.2)	−0.1 (−0.2 to −0.1)
Left atrial global longitudinal strain (%)	30.1 ± 3.5	26.9 ± 2.5	29.7 ± 4.0	35.0 ± 2.4*^,†,‡^	−3.1 (−4.0 to −2.2)	5.3 (4.2 to 6.4)
Mitral regurgitation volume (mL)	24.3 ± 10.0	25.1 ± 10.3	23.6 ± 9.1	22.7 ± 9*^,†^	0.4 (0.4 to 1.1)	−0.5 (−1.3 to −0.5)
Effective regurgitant orifice area (cm^2^)	0.2 ± 0.1	0.2 ± 0.1	0.2 ± 0.1	0.2 ± 0.1	−0.005 (−0.029 to 0.011)	−0.001 (−0.007 to 0.004)
Estimated pulmonary artery systolic pressure (mmHg)	43.3 ± 6.6	46.0 ± 6.8	42.8 ± 10.6	41.7 ± 9.9*^,†,‡^	2.7 (1.9 to 3.5)	−1.2 (−2.4 to 0.1)

Data are expressed as mean ± standard deviation.

* *p* < 0.05 interaction, ^†^*p* < 0.05 vs. before, ^‡^*p* < 0.05 vs. control groups.

SI, stroke volume index; CI, cardiac output index; E, Peak early flow velocity; A, Late diastolic flow velocity; E/A, Ratio of peak early and late diastolic flow velocities; DcT, deceleration time; e′, Peak early diastolic tissue velocity; E/e', Ratio of the mitral inflow early diastolic velocity to the mean e′ velocity from the septal and lateral sides of the mitral annulus.

### 3.6. Impact of exercise training intervention on hemodynamic indicators during exercise

After the exercise training intervention, remarkable interactions were observed in peak watt, peak SI, peak HR, peak CI, peak a-vO_2_ diff, peakVO_2_/HR, and HRR as hemodynamic indicators (Table 4). In addition, notable interactions were observed in VO_2_, work rate, and hemodynamic indicators during AT, with marked improvements in the intervention group.

**Table 4:** Changes in cardiorespiratory exercise testing and hemodynamics data between both groups before and after cardiac rehabilitation intervention.

	Control group (*n* = 50)		Intervention group (*n* = 49)		Change in 5 months, mean within-group difference (95% CI)	
Parameters	Baseline	Follow-up	Baseline	Follow-up	Control group (*n* = 50)	Intervention group (*n* = 49)
Resting (Sitting posture)						
VO_2_ (mL/min/kg)	3.3 ± 0.6	3.6 ± 0.7	3.2 ± 0.5	3.7 ± 0.7*^,†^	0.2 (0.1 to 0.3)	0.5 (0.3 to 0.6)
SI (mL/m^2^)	36.1 ± 3.6	36.5 ± 4.0	33.7 ± 4.8	36.7 ± 6.2*^,†^	0.4 (−0.3 to 1.1)	3.0 (1.9 to 4.2)
HR (bpm/min)	72 ± 4	75 ± 6	74 ± 7	74 ± 7*	3.6 (2.3 to 4.9)	0.4 (0.3 to 1.2)
CI (L/min/m^2^)	2.6 ± 0.3	2.8 ± 0.4	2.5± 0.4	2.7± 0.5	0.2 (0.1 to 0.2)	0.2 (0.1 to 0.3)
a-vO_2_ diff (mL/100 mL)	5.1 ± 0.8	5.2 ± 0.8	5.1 ± 0.5	5.3 ± 0.5	0.1 (0.1 to 0.2)	0.1 (0.1 to 0.2)
VO_2_/HR (mL/beat)	3.2 ± 0.6	3.3 ± 0.6	3.0± 0.5	3.3± 0.5*^,†^	0.1 (0.1 to 0.2)	0.3 (0.2 to 0.4)
Anaerobic threshold						
VO_2_ (mL/min/kg)	10.3 ± 2.7	9.6 ± 2.4	9.6 ± 2.0	11.0 ± 2.4*^,†,‡^	−0.7 (−0.9 to −0.4)	1.4 (1.2 to 1.6)
Work rate (watt)	57 ± 15	56 ± 13	54 ± 11	65 ± 16*^,†,‡^	−1.8 (−2.8 to −0.7)	11 (9 to 13)
SI (mL/m^2^)	44.1 ± 3.9	42.3 ± 2.9	42.5 ± 3.0	43.8 ± 3.1*^,†,‡^	−1.8 (−2.5 to −1.1)	1.3 (1.1 to 1.5)
HR (bpm/min)	104 ± 11	99 ± 7	108 ± 8	111 ± 9*^,†,‡^	−5.2 (−7.3 to −3.2)	4 (2 to 5)
CI (L/min/m^2^)	4.6 ± 0.5	4.2 ± 0.4	4.6 ± 0.5	4.9 ± 0.5*^,†,‡^	−0.4 (−0.5 to −0.3)	0.3 (0.2 to 0.4)
a-vO_2_ diff (mL/100 mL)	8.7 ± 1.4	9.1 ± 1.5	8.3 ± 1.2	8.8 ± 1.4*^,^^†^	0.3 (0.2 to 0.5)	0.5 (0.4 to 0.6)
VO_2_/HR (mL/beat)	6.7 ± 1.4	6.7 ± 1.4	6.2 ± 1.3	6.7 ± 1.5*^,†^	0.1 (−0.1 to 0.2)	0.5 (0.5 to 0.6)
Peak exercise						
Work rate (watt)	79 ± 24	70 ± 25	76 ± 21	83 ± 27*^,†,‡^	−9 (−11 to −7)	7 (5 to 10)
SI (mL/m^2^)	44.2 ± 4.1	42.9 ± 4.2	42.6 ± 4.0	42.8 ± 3.9*	−1.2 (−1.5 to −1.0)	0.2 (−0.1 to 0.6)
HR (bpm/min)	125 ± 14	120 ± 15	127 ± 13	131 ± 15*^,†,‡^	−5 (−6 to −4)	4 (3 to 6)
CI (L/min/m^2^)	5.6 ± 1.0	5.2 ± 1.0	5.4 ± 0.9	5.6 ± 1.0*^,†,‡^	−0.4 (−0.4 to −0.3)	0.2 (0.1 to 0.3)
a-vO_2_ diff (mL/100 mL)	10.0 ± 1.6	9.6 ± 1.8	10.0 ± 1.5	10.2 ± 1.6*^,†,‡^	−0.5 (−0.6 to −0.4)	0.3 (0.2 to 0.3)
VO_2_/HR (mL/beat)	7.7 ± 1.5	7.2 ± 1.6	7.4 ± 1.7	7.6 ± 1.7*^,†^	−0.5 (−0.6 to −0.4)	0.2 (0.1 to 0.3)
Other indicators						
*Δ*VO_2_/*Δ*Work rate (mL/min/work rate)	8.0 ± 0.7	7.5 ± 0.6	8.0 ± 0.9	8.5 ± 0.6*^,†,‡^	−0.6 (−0.6 to −0.4)	0.5 (0.3 to 0.6)
Minimum VE/VCO_2_ (mL/mL)	34.4 ± 2.5	36.2 ± 2.0	34.3 ± 2.4	30.9 ± 2.5*^,†,‡^	1.8 (1.3 to 2.3)	−3.4 (−3.8 to −2.9)
Percent of peak HR (%)	86 ± 9	82 ± 10	87 ± 8	90 ± 10*^,†,‡^	−3.5 (−4.2 to −2.7)	3 (2 to 4)
HRR (beat)	8 ± 3	7 ± 2	9 ± 3	11 ± 4*^,†,‡^	−1 (−2 to −1)	2 (1 to 3)

Data are expressed as mean ± standard deviation.

* *p* < 0.05 interaction, ^†^*p* < 0.05 vs. before, ^‡^*p* < 0.05 vs. control groups.

RER, Respiratory exchange ratio; VO_2_, oxygen uptake; SI, Stroke volume index; HR, Heart rate; CI, Cardiac output index; a-vO_2_ diff, arterial-venous oxygen difference; VO_2_/HR, Oxygen pulse; *Δ*VO_2_/*Δ*Work rate, oxygen uptake-work rate relationship; Minimum VE/VCO_2_, minimum ventilatory equivalent for carbon dioxide; VE vs. VCO_2_ slope, ventilatory equivalent vs. carbon dioxide output slope. .

### 3.7. Occurrence of adverse events associated with exercise training intervention

Adverse events associated with exercise training interventions occurred in 22% of the patients in the intervention group (Table 5). The adverse events that occurred were arrhythmia during exercise training in 6% of cases (3 cases), fatigue with a Borg scale rating >15 in 4% of cases (2 cases), and skeletal muscle pain after exercise in 12% of cases (6 cases).

**Table 5:** Occurrence of adverse events associated with exercise training intervention.

Characteristics	Intervention group (*n* = 49)	Control group (*n* = 50)
Arrhythmia during exercise training (%)	6	-
Fatigue over Borg scale 15 (%)	4	-
Skeletal muscle pain after exercise (%)	12	-

Nominal variables are expressed as percentages.

## 4. Discussion

We had four major findings in this intervention trial on older Japanese patients with strictly diagnosed HFpEF. First, exercise training improved the exercise capacity indices of peakVO_2_ and AT-VO_2_. Second, the intervention group with a 10% improvement in peakVO_2_ from baseline had a considerably lower prevalence of diabetes mellitus, a lower VE vs. VCO_2_ slope, and considerably higher LA-GLS values than the group without improvement. Third, exercise training improved the following hemodynamic indices: VE vs. VCO_2_ slope, peak CI, peak SI, peak HR, peak a-vO_2_ diff, and HRR. Finally, exercise training improved LA-GLS and LV-GLS as measured by echocardiography. To the best of our knowledge, this is the first study to evaluate the effects of an exercise training intervention on peakVO_2_, its predictors, and LA and LV structural function in older Japanese patients with HFpEF aged >70 years. Our study was not randomized or blinded; however, the primary endpoint was not significantly different between the groups at baseline. Thus, results obtained without escalating the dose of antidiabetic drugs such as sodium-glucose cotransporter 2 inhibitors have important clinical significance. Exercise capacity is a determinant of prognosis in HFpEF, and exercise training is one of the few interventions used to improve exercise intolerance in patients with HFpEF.

### 4.1. Effects of exercise training interventions on peakVO_2_ in older HFpEF patients

In our study, the intervention among older Japanese patients with HFpEF led to a substantial mean improvement of 8.3% in peakVO_2_ compared to the Control group. This improvement was associated with a significant mean difference of −20.6%, supported by a 95% confidence interval of −23.4% to −17.9%, with a *p*-value of less than 0.001, all measured relative to baseline. Although many studies have reported that exercise training interventions improve peakVO_2_ in patients with HFpEF (8, 9), there is a distinct paucity of reports on East Asian patients with HFpEF. Our study is possibly the first to demonstrate that an exercise training intervention improves peakVO_2_, a prognostic factor in older Japanese patients with HFpEF aged >70 years. Compared to the study by Fu et al. (12) on East Asian patients with HFpEF of the same ethnicity, our study participants were older, had various comorbidities, and had severe baseline exercise intolerance. We extended previous findings by examining the effects of exercise training interventions on peakVO_2_ in patients with the common clinical symptoms of HFpEF. However, there are some caveats to the results of our study. Although 31% of the intervention group achieved a clinically meaningful improvement of 10% in peakVO_2_ from baseline, the improved peakVO_2_ resulting from the exercise training intervention combined with AT-intensity aerobic exercise and strength training was less drastic in this study than in a similar exercise-style intervention study in Caucasian patients with HFpEF, which reported an average 16% improvement in peakVO_2_ (16). The reason for this variation is unclear, as the age, sex distributions, and diuretic treatment were similar in both studies. However, the prevalence of diabetes mellitus (51% vs. 10%) and the proportion of patients falling under NYHA functional class III were higher (49% vs. 20%), and the mean peakVO_2_ at baseline was lower (13.8 vs. 16.1 ml/min/kg) in our study. In patients with HF, diabetes mellitus complications reportedly reduce the degree of improvement in peakVO_2_ induced by exercise training (43). We cannot rule out the possibility that our study participants had more severe HF than those in the study by Edelmann et al. (16). Therefore, it is possible that various complications and their severity according to the NYHA classification affected the peakVO_2_ improvement. Further studies are required to examine the effects of exercise training on various complications and cardiovascular phenotypes.

### 4.2. Clinical characteristics of the groups with a clinically meaningful change in peakVO_2_ from baseline

As shown in Table 2, no significant differences in age, sex, and indicators related to the severity of HF (e.g., NYHA functional classification, BNP, mitral regurgitation, and estimated pulmonary artery systolic pressure) were found between the improvement and non-improvement groups. The most significant difference between the two groups was the prevalence of diabetes mellitus. Since both groups had similar exercise styles and intensities, diabetes mellitus was a factor closely related to the improvement in peakVO_2_ due to exercise training. However, reports examining the exercise capacity and improvement response to exercise training in patients with HFpEF and diabetes mellitus are limited; therefore, the underlying mechanisms remain largely unknown. In a previous study, we reported that HFpEF complicated with diabetes mellitus exhibits an additive decrease in peakVO_2_ and hemodynamic response during exercise (44). Furthermore, insights from the sub-analysis of the Heart Failure–A Controlled Trial Investigating Outcomes of Exercise TraiNing (HF-ACTION) trial show that diabetes mellitus complications reportedly reduce the degree of improvement in peakVO_2_ induced by exercise training (43). These reports partially support our findings. In the HF-ACTION trial targeting HF with reduced ejection fraction, it was revealed that comorbid diabetes mellitus inhibits the improvement of peakVO_2_ by exercise training; however, this is the first finding to indicate that a similar phenomenon may occur in patients with HFpEF. Additionally, the HF-ACTION trial indicated that slower improvement in exercise capacity in patients with diabetes mellitus may be caused by a combination of poor adherence, high BMI, and physiological maladaptation (43); however, this has not been investigated in patients with HFpEF. Therefore, collecting more cases and conducting further studies are necessary to clarify the effects of exercise training on peakVO_2_ and its predictors in patients with HFpEF and diabetes mellitus in the future.

### 4.3. Effects of exercise training intervention on hemodynamics and predictors of peakVO_2_ improvement

Exercise training in older patients with HFpEF not only extensively improved peak SI, peak HR, and peak a-vO_2_ diff, which are the components of Fick's equation, but also considerably improved HRR, a cardiac autonomic index, and VE vs. VCO_2_ slope, an index of ventilation efficiency.

Multiple studies have shown that exercise training interventions are only effective in maintaining peak SI in patients with HFpEF (12, 45). Our results also showed a statistically significant (*p* < 0.05) difference due to the interaction; however, the improvement rate associated with the exercise training intervention was only an average of 0.6%. Based on this, it can be said that the exercise training intervention for peak SI shows only a maintenance effect, and the results are similar to those of previous studies on Western populations.

Peak HR, an index of HR response during exercise and one of the components of Fick's equation, was most improved with the exercise training intervention. Multiple study results to date are neutral regarding the improvement in HR response associated with exercise training interventions (12, 46). However, our study showed that HRR, a cardiac autonomic function index that controls the HR response during exercise, was also markedly improved by the exercise training intervention. Several intervention studies (46, 47) reported that chronotropic incompetence and HRR were improved by exercise therapy alone. Therefore, we cannot exclude the possibility that exercise training had a positive effect on the HR response by improving cardiac autonomic nerve function. The SV reached a plateau at 40%–50% of peak exercise, and subsequently, the increasing HR led to an increase in CO to oxygenate peripheral tissues (48). Furthermore, the cardiac sympathetic nervous system (HR response) is involved in 60% of the exercise load after peak exercise (49). Thus, an exercise training intervention with an improved HR response during exercise is important for improving exercise intolerance in patients with HFpEF. Improvements in the prevalence of peak HR, HRR, and chronotropic incompetence in patients with HFpEF aged over 70 years without treatment with *β*-blockers underscore the benefits of exercise training on peakVO_2_ and its predictors. Furthermore, peak a-vO_2_ diff, an index that partially reflects the oxygen extraction capacity of peripheral tissues, also improved by a mean of 2.9% after the exercise training intervention. Tucker et al. (50) reported that the presumed a-vO_2_ diff as a peripheral mechanism contributed greatly to the improvement in peakVO_2_ after exercise training in patients with HFpEF. The results of these studies support our findings. However, as the proportion of patients with diabetes in this study was high (approximately 50%), the high contribution of the improvement in cardiac autonomic neuropathy associated with exercise training cannot be ruled out.

The contribution of Fick's equation to exercise intolerance in patients with HFpEF has been the focus of debate over the past few years. However, to fully understand exercise intolerance in patients with HFpEF, further integrated studies on hemodynamics are needed. Exercise training extensively improved the VE vs. VCO_2_ slope, which is an index of ventilation efficiency during exercise. The VE vs. VCO_2_ slope is also an index of pulmonary artery blood flow and ventilation/perfusion imbalance, and high values in patients with HFpEF are reportedly associated with survival prognosis (51). In addition, our results showed that the group that achieved a 10% improvement in peakVO_2_ from baseline with exercise training had a markedly lower VE vs. VCO_2_ slope. Therefore, the favorable changes in VE vs. VCO_2_ slope caused by exercise training in this study are clinically significant. To date, no study, including a meta-analysis based on four studies, has found any improvement in VE/VCO_2_ with exercise training. However, the study by Fu et al. (12) is one of the few to report such improvements in VE/VCO_2_. Although the underlying mechanism is unknown, cardiac autonomic neuropathy may exacerbate the ventilatory response to exercise by excessively increasing the respiratory rate and alveolar ventilation (52). In our study, exercise training improved circulatory responses such as cardiac autonomic neuropathy, HRR, and peak CI during exercise. Therefore, improvement of ventilation/perfusion imbalance and cardiac autonomic neuropathy associated with insufficient cardiac output in HFpEF may have caused the decrease in the VE vs. VCO_2_ slope.

### 4.4. Effects of exercise training intervention on echocardiography data

In this study, exercise training considerably improved the LA and LV-GLS. A meta-analysis (53) reported that exercise training markedly improved LV-GLS in patients with cardiovascular disease, but data on HFpEF patients aged >70 years were lacking. We extended the previous findings by demonstrating the effects of exercise training on LV-GLS in older patients with HFpEF and various comorbidities. These findings are clinically important, as reduced LV-GLS and LA-GLS are strong prognostic indicators of future cardiovascular dysfunction, exercise capacity, and mortality (54–56). The E/e′, LVM, and LVEDV indices were not significantly different from the baseline after exercise training. These results are consistent with those reported by Kitzman et al. (7). However, despite the moderate-intensity continuous training with resistance training employed in this study, our results are different from those reported by Edelmann et al. (16) with a similar exercise-style intervention. The E/e′ ratio at baseline was more severe in our study, with a mean of 14.5. Similar to peakVO_2_, the effect of exercise training on cardiovascular function may be considerably influenced by the baseline severity and prevalence of DM. Further studies, such as a subgroup analysis with an increased number of cases, are needed.

### 4.5. Limitations

In this study, an open-label, non-randomized control design is the major limitation. In addition, selection bias could not be completely ruled out because it was a single-center study. Moreover, this study included only Japanese individuals, who differ from Caucasians in terms of ethnicity and physique. Previous studies have included different ethnicities. Impedance cardiography, a non-invasive method for assessing CO, is highly correlated with the direct Fick method in healthy individuals. However, it has been reported that the SV may be overestimated when patients with HF are included as participants (57). Therefore, errors may have occurred during the measurements in participants with the same HF symptoms. However, our study participants had a more preserved LVEF than those in the study by Kemps et al. (57); furthermore, patients with ischemic and dilated cardiomyopathy were not included, and their clinical characteristics were notably different. A stress test that combines CPET and echocardiography showed clinically acceptable measurement accuracy, which is consistent with the CO value measured directly by Fick during exercise. Further, various types of information can be obtained during exercise (e.g., LV-GLS, E/e′, and LVEF). This may provide a compatible alternative to the direct Fick method (58).

## 5. Conclusions

Exercise training interventions in older patients strictly diagnosed with HFpEF aged ≥70 years improved peakVO_2_, hemodynamic indices and some echocardiography indices. Furthermore, our results clarified the clinical characteristics of the group that achieved a clinically meaningful improvement of 10% in peakVO_2_ from baseline. These results suggest the benefits of exercise training in patients with HFpEF aged older than 70 years and that intervention strategies for patients with HFpEF and diabetes mellitus need reconsideration.

## Data Availability

The raw data supporting the conclusions of this article will be made available by the authors, without undue reservation.
