# Correction: Exercise training affects hemodynamics and exercise capacity in cases of heart failure with preserved ejection fraction: a non-randomized controlled trial in individuals aged 65–80 years

**DOI:** 10.3389/fcvm.2026.1806631

**Published:** 2026-03-30

**Authors:** Yousuke Sugita, Katsuhiko Ito, Yui Yoshioka, Ayano Kudo, Sota Arakawa, Satoshi Sakai

**Affiliations:** 1 Faculty of Health Sciences, Tsukuba University of Technology, Tsukuba, Japan; 2 Department of Rehabilitation, National Hospital Organization Matsumoto National Hospital, Matsumoto, Japan; 3 Department of Rehabilitation, Musashino General Hospital, Kawagoe, Japan

**Keywords:** echocardiography, exercise training, heart failure with preserved ejection fraction, peak arteriovenous oxygen difference, peak heart rate, peak oxygen uptake, peak stroke volume, ventilatory equivalent vs. carbon dioxide output slope

There was a mistake in figure 2, table 3 and table 4 as published. These errors were due to typographical and transcription errors in the presentation of the results. The corrected figure 2, table 3 and table 4 appear below.

**Figure 2:**
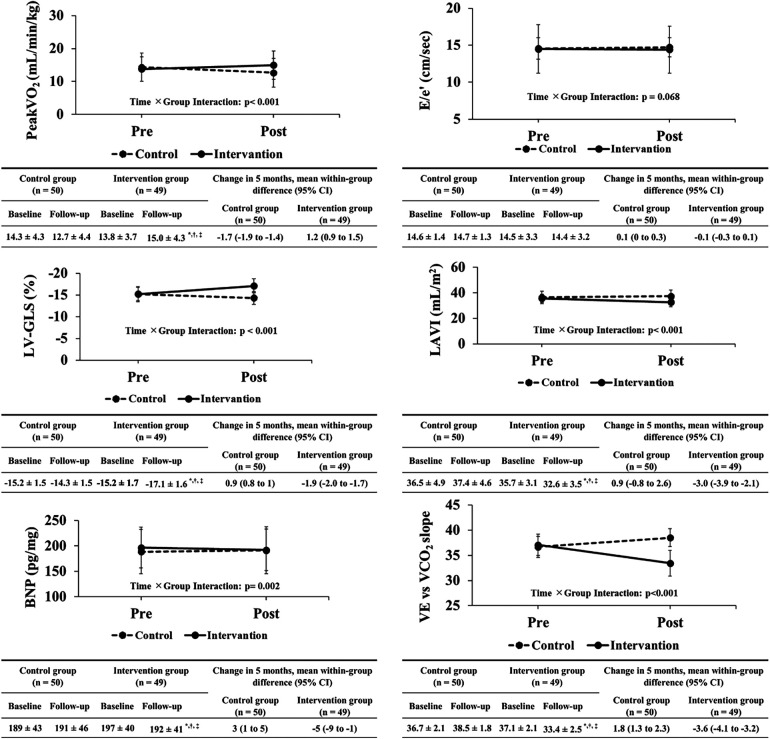
Changes in primary and secondary outcomes between both groups before and after exercise training intervention. peakVO_2_, peak oxygen uptake; E/e′, ratio of the mitral inflow early diastolic velocity to the mean e′ velocity from the septal and lateral sides of the mitral annulus; e′, peak early diastolic tissue velocity; LV-GLS, left ventricular global longitudinal strain; LAVI, left atrial volume index; BNP, brain natriuretic peptide; VE vs. VCO_2_ slope, ventilatory equivalent vs. carbon dioxide output slope.

In Figure 2, the incorrect follow-up values were corrected for peakVO_2_ in the control group, E/e′ in the intervention group, and LV-GLS in the control group. The graphical trends and statistical analyses were correct in the published article.

**Table 3:** Changes in echocardiography data between both groups before and after cardiac rehabilitation intervention.

	Control group (*n* = 50)		Intervention group (*n* = 49)		Change in 5 months, mean within-group difference (95% CI)	
Parameters	Baseline	Follow-up	Baseline	Follow-up	Control group (*n* = 50)	Intervention group (*n* = 49)
Epicardial adipose tissue thickness (mm)	8.4 ± 0.9	9.0 ± 1.0	8.4 ± 0.6	7.5 ± 0.6*^,^^†,‡^	0.6 (0.5 to 0.7)	−0.8 (−0.8 to −0.9)
Interventricular septal thickness at end diastole (mm)	10.1 ± 1.0	10.2 ± 1.0	10.0 ± 0.9	10.0 ± 0.9*	0.11 (0.09 to 0.14)	0.02 (−0.02 to 0.07)
Posterior wall thickness at end diastole (mm)	10.2 ± 1.1	10.3 ± 1.1	10.0 ± 0.9	10.0 ± 0.9*	0.1 (0.1 to 0.2)	−0.002 (−0.029 to 0.033)
Left ventricular end-diastolic diameter (mm)	46.1 ± 1.9	46.0 ± 1.8	45.9 ± 3.0	45.8 ± 2.8	−0.1 (−0.2 to 0.1)	−0.13 (−0.29 to 0.03)
Left ventricular end-systolic diameter (mm)	29.6 ± 2.3	29.8 ± 2.4	28.9 ± 3.4	28.9 ± 3.4*	0.2 (0.1 to 0.3)	0.02 (−0.02 to 0.06)
Left ventricular end-diastolic volume index (mL/m^2^)	56.7 ± 6.1	56.2 ± 6.1	56.5 ± 9.5	56.4 ± 8.4	−0.5 (−1.4 to 0.3)	−0.1 (−1.3 to 1.2)
Left ventricular end-systolic volume index (mL/m^2^)	19.8 ± 3.8	20.0 ± 3.9	18.7 ± 5.0	18.9 ± 5.3	0.3 (−1.4 to 0.3)	0.2 (−0.2 to 0.7)
Left ventricular ejection fraction (%)	65.0 ± 6.0	64.3 ± 6.2	66.5 ± 8.2	66.3 ± 8.3	−0.7 (−1.2 to −0.3)	−0.2 (−0.6 to 0.1)
Left atrial ejection fraction (%)	47.5 ± 6.1	47.2 ± 6.5	45.7 ± 4.2	46.9 ± 6.2	−0.3 (−3.0 to 2.4)	1.3 (−0.5 to 3.0)
SI (mL/m^2^)	36.9 ± 5.5	36.1 ± 5.4	37.8 ± 8.8	37.5 ± 7.8	−0.8 (−1.4 to −0.1)	−0.3 (−1.2 to 0.6)
CI (L/min/m^2^)	2.5 ± 0.4	2.4 ± 0.4	2.7 ± 0.7	2.5 ± 0.5	−0.1 (−0.2 to −0.1)	−0.2 (−0.3 to −0.2)
Left ventricular mass index (g/m^2^)	117 ± 16	118 ± 18	115 ± 20	115 ± 20	1.1 (−0.8 to 3.0)	0.6 (−1.9 to 3.1)
Relative wall thickness	0.44 ± 0.05	0.45 ± 0.05	0.44 ± 0.04	0.44 ± 0.04*	0.006 (0.003 to 0.008)	0.001 (−0.001 to 0.003)
E (cm/sec)	59.7 ± 9.1	58.0 ± 9.4	60.9 ± 10.8	62.4 ± 10.6*^,†,‡^	−1.7 (−2.3 to −1.2)	1.4 (1.2 to 1.7)
A (cm/sec)	77.0 ± 13.1	74.9 ± 13.3	76.9 ± 7.1	77.5 ± 6.9*^,†^	−2.1 (−2.7 to −1.5)	0.6 (0.5 to 0.8)
E/A	0.8 ± 0.1	0.8 ± 0.1	0.8 ± 0.1	0.8 ± 0.1*^,†^	−0.001 (−0.008 to 0.006)	0.012 (0.011 to 0.015)
DcT (cm/sec)	236 ± 24.8	248 ± 24	241 ± 26	237 ± 25*^,†,‡^	11.6 (10.1 to 13.0)	−4.2 (−5.4 to −3.1)
Lateral e′ (cm/sec)	5.4 ± 1.1	5.1 ± 1.1	5.5 ± 2.0	5.6 ± 2*^,†^	−0.3 (−0.3 to −0.2)	0.1 (0.1 to 0.2)
Medial e′ (cm/sec)	2.9 ± 1.0	2.9 ± 1.1	3.6 ± 1.5	3.7 ± 1.5*^,†,‡^	−0.1 (−0.1 to 0.03)	0.1 (0.05 to 0.1)
Mean e′ (cm/sec)	4.2 ± 1.0	4.0 ± 1.1	4.5 ± 1.7	4.7 ± 1.7*^,†,‡^	−0.2 (−0.2 to −0.1)	0.1 (0.1 to 0.2)
E/e′ (cm/sec)	14.6 ± 1.4	14.7 ± 1.3	14.5 ± 3.3	14.4 ± 3.2	0.1 (−0.03 to 0.3)	−0.1 (−0.3 to 0.1)
Peak tricuspid regurgitation velocity (m/s)	2.8 ± 0.3	3.0 ± 0.3	2.8 ± 0.5	2.8 ± 0.5*^,†,‡^	0.1 (0.1 to 0.2)	−0.1 (−0.2 to −0.1)
Left atrial global longitudinal strain (%)	30.1 ± 3.5	26.9 ± 2.5	29.7 ± 4.0	35.0 ± 2.4*^,†,‡^	−3.1 (−4.0 to −2.2)	5.3 (4.2 to 6.4)
Mitral regurgitation volume (mL)	24.3 ± 10.0	25.1 ± 10.3	23.6 ± 9.1	22.7 ± 9*^,†^	0.4 (0.4 to 1.1)	−0.5 (−1.3 to −0.5)
Effective regurgitant orifice area (cm^2^)	0.2 ± 0.1	0.2 ± 0.1	0.2 ± 0.1	0.2 ± 0.1	−0.005 (−0.029 to 0.011)	−0.001 (−0.007 to 0.004)
Estimated pulmonary artery systolic pressure (mmHg)	43.3 ± 6.6	46.0 ± 6.8	42.8 ± 10.6	41.7 ± 9.9*^,†,‡^	2.7 (1.9 to 3.5)	−1.2 (−2.4 to 0.1)

Data are expressed as mean ± standard deviation.

* *p* < 0.05 interaction, ^†^*p* < 0.05 vs. before, ^‡^*p* < 0.05 vs. control groups.

SI, stroke volume index; CI, cardiac output index; E, Peak early flow velocity; A, Late diastolic flow velocity; E/A, Ratio of peak early and late diastolic flow velocities; DcT, deceleration time; e′, Peak early diastolic tissue velocity; E/e', Ratio of the mitral inflow early diastolic velocity to the mean e′ velocity from the septal and lateral sides of the mitral annulus.

In Table 3, the follow-up value for left ventricular end-diastolic volume index (LVEDVI) in the control group contained a transcription error and has been corrected.

**Table 4:** Changes in cardiorespiratory exercise testing and hemodynamics data between both groups before and after cardiac rehabilitation intervention.

	Control group (*n* = 50)		Intervention group (*n* = 49)		Change in 5 months, mean within-group difference (95% CI)	
Parameters	Baseline	Follow-up	Baseline	Follow-up	Control group (*n* = 50)	Intervention group (*n* = 49)
Resting (Sitting posture)						
VO_2_ (mL/min/kg)	3.3 ± 0.6	3.6 ± 0.7	3.2 ± 0.5	3.7 ± 0.7*^,†^	0.2 (0.1 to 0.3)	0.5 (0.3 to 0.6)
SI (mL/m^2^)	36.1 ± 3.6	36.5 ± 4.0	33.7 ± 4.8	36.7 ± 6.2*^,†^	0.4 (−0.3 to 1.1)	3.0 (1.9 to 4.2)
HR (bpm/min)	72 ± 4	75 ± 6	74 ± 7	74 ± 7*	3.6 (2.3 to 4.9)	0.4 (0.3 to 1.2)
CI (L/min/m^2^)	2.6 ± 0.3	2.8 ± 0.4	2.5± 0.4	2.7± 0.5	0.2 (0.1 to 0.2)	0.2 (0.1 to 0.3)
a-vO_2_ diff (mL/100 mL)	5.1 ± 0.8	5.2 ± 0.8	5.1 ± 0.5	5.3 ± 0.5	0.1 (0.1 to 0.2)	0.1 (0.1 to 0.2)
VO_2_/HR (mL/beat)	3.2 ± 0.6	3.3 ± 0.6	3.0± 0.5	3.3± 0.5*^,†^	0.1 (0.1 to 0.2)	0.3 (0.2 to 0.4)
Anaerobic threshold						
VO_2_ (mL/min/kg)	10.3 ± 2.7	9.6 ± 2.4	9.6 ± 2.0	11.0 ± 2.4*^,†,‡^	−0.7 (−0.9 to −0.4)	1.4 (1.2 to 1.6)
Work rate (watt)	57 ± 15	56 ± 13	54 ± 11	65 ± 16*^,†,‡^	−1.8 (−2.8 to −0.7)	11 (9 to 13)
SI (mL/m^2^)	44.1 ± 3.9	42.3 ± 2.9	42.5 ± 3.0	43.8 ± 3.1*^,†,‡^	−1.8 (−2.5 to −1.1)	1.3 (1.1 to 1.5)
HR (bpm/min)	104 ± 11	99 ± 7	108 ± 8	111 ± 9*^,†,‡^	−5.2 (−7.3 to −3.2)	4 (2 to 5)
CI (L/min/m^2^)	4.6 ± 0.5	4.2 ± 0.4	4.6 ± 0.5	4.9 ± 0.5*^,†,‡^	−0.4 (−0.5 to −0.3)	0.3 (0.2 to 0.4)
a-vO_2_ diff (mL/100 mL)	8.7 ± 1.4	9.1 ± 1.5	8.3 ± 1.2	8.8 ± 1.4*^,^^†^	0.3 (0.2 to 0.5)	0.5 (0.4 to 0.6)
VO_2_/HR (mL/beat)	6.7 ± 1.4	6.7 ± 1.4	6.2 ± 1.3	6.7 ± 1.5*^,†^	0.1 (−0.1 to 0.2)	0.5 (0.5 to 0.6)
Peak exercise						
Work rate (watt)	79 ± 24	70 ± 25	76 ± 21	83 ± 27*^,†,‡^	−9 (−11 to −7)	7 (5 to 10)
SI (mL/m^2^)	44.2 ± 4.1	42.9 ± 4.2	42.6 ± 4.0	42.8 ± 3.9*	−1.2 (−1.5 to −1.0)	0.2 (−0.1 to 0.6)
HR (bpm/min)	125 ± 14	120 ± 15	127 ± 13	131 ± 15*^,†,‡^	−5 (−6 to −4)	4 (3 to 6)
CI (L/min/m^2^)	5.6 ± 1.0	5.2 ± 1.0	5.4 ± 0.9	5.6 ± 1.0*^,†,‡^	−0.4 (−0.4 to −0.3)	0.2 (0.1 to 0.3)
a-vO_2_ diff (mL/100 mL)	10.0 ± 1.6	9.6 ± 1.8	10.0 ± 1.5	10.2 ± 1.6*^,†,‡^	−0.5 (−0.6 to −0.4)	0.3 (0.2 to 0.3)
VO_2_/HR (mL/beat)	7.7 ± 1.5	7.2 ± 1.6	7.4 ± 1.7	7.6 ± 1.7*^,†^	−0.5 (−0.6 to −0.4)	0.2 (0.1 to 0.3)
Other indicators						
*Δ*VO_2_/*Δ*Work rate (mL/min/work rate)	8.0 ± 0.7	7.5 ± 0.6	8.0 ± 0.9	8.5 ± 0.6*^,†,‡^	−0.6 (−0.6 to −0.4)	0.5 (0.3 to 0.6)
Minimum VE/VCO_2_ (mL/mL)	34.4 ± 2.5	36.2 ± 2.0	34.3 ± 2.4	30.9 ± 2.5*^,†,‡^	1.8 (1.3 to 2.3)	−3.4 (−3.8 to −2.9)
Percent of peak HR (%)	86 ± 9	82 ± 10	87 ± 8	90 ± 10*^,†,‡^	−3.5 (−4.2 to −2.7)	3 (2 to 4)
HRR (beat)	8 ± 3	7 ± 2	9 ± 3	11 ± 4*^,†,‡^	−1 (−2 to −1)	2 (1 to 3)

Data are expressed as mean ± standard deviation.

* *p* < 0.05 interaction, ^†^*p* < 0.05 vs. before, ^‡^*p* < 0.05 vs. control groups.

RER, Respiratory exchange ratio; VO_2_, oxygen uptake; SI, Stroke volume index; HR, Heart rate; CI, Cardiac output index; a-vO_2_ diff, arterial-venous oxygen difference; VO_2_/HR, Oxygen pulse; *Δ*VO_2_/*Δ*Work rate, oxygen uptake-work rate relationship; Minimum VE/VCO_2_, minimum ventilatory equivalent for carbon dioxide; VE vs. VCO_2_ slope, ventilatory equivalent vs. carbon dioxide output slope.

In Table 4, across the table, the values in the “Change (within-group difference)” column were incorrectly assigned between the control and intervention groups. The baseline and follow-up values were correct, and only the change values have been corrected.

The original version of this article has been updated.

