# Supplementary material for: Exercise training affects hemodynamics and exercise capacity in cases of heart failure with preserved ejection fraction: a non-randomized controlled trial in individuals aged 65–80 years
Source: Front Cardiovasc Med. 2023 Oct 30;10:1246739. doi: 10.3389/fcvm.2023.1246739 (PMC10646767; doi:10.3389/fcvm.2023.1246739)
Supplement: Supplementary file 1 [file Datasheet1.docx]

Supplementary Material

Exercise training affects hemodynamics and exercise capacity in cases of heart failure with preserved ejection fraction: A non-randomized controlled trial in individuals aged 65–80 years

**Yousuke Sugita*, Katsuhiko Ito, Yui Yoshioka, Shigeki Sakurai, Ayano Kudo, Sota Arakawa, Satoshi Sakai**

*** Correspondence:** Yousuke Sugita, [y.sugita@cc.k.tsukuba-tech.ac.jp](mailto:y.sugita@cc.k.tsukuba-tech.ac.jp)

# Supplementary Data

**1. Supplementary Materials**

BMI (kg/m^2^) = body weight (kg) / height^2^ (m)

BSA (m^2^) = body weight^0.425^ × height^0.725^ × 0.007184

eGFR (mL/min/1.73 m^2^) = [104 × cystatin-c^-1.019^ × 0.996^age^ × 0.929 (if female)] − 8

HOMA-IR = fasting insulin (U/mL) × fasting glucose (mg/dL)/405

SV (mL) = LVEDV (mL) – LVESV (mL)

LVEF (%) = SV / LVEDV (mL) × 100

LAVI (mL/m^2^) = LAV max / BSA (m^2^)

LAEF (%) = (LAV max – LAV min) / LAV max × 100

Estimated pulmonary artery systolic pressure (mmHg) = 4 × (tricuspid regurgitation velocity)^2^ + estimated right atrial pressure

HRR = peak heart rate or HR – HR 1 min after the end of the exercise load.

VO_2_/HR (mL/beat) = VO_2_ (mL) / HR (beats/min)

CO (L/min) = SV (mL/min) × HR (beats/min)

a-vO_2_ (mL/100 mL) = CO (L) / VO_2_ (mL)

**2. Materials and Methods**

**2.1 Study design and participants**

Between 2016 and 2021, 117 individuals who did not meet the exclusion criteria were enrolled in the study, and 99 were included in the final analysis. All study participants showed predicted peakVO2 ≤ 80% as evidence of obvious exercise intolerance. The exclusion criteria for participants in this study were as follows: exacerbation of subjective symptoms of heart failure (including dyspnea and fatigue) within the past week; unstable angina or low threshold (induced by slow walking on level ground, two metabolic equivalents); severe valvular disease, for which surgery is indicated (especially aortic stenosis); severe left ventricular outflow tract stenosis (obstructive hypertrophic cardiomyopathy); untreated exercise-induced severe arrhythmia (ventricular fibrillation and persistent ventricular tachycardia); active myocarditis; acute systemic disease or fever; other diseases, for which exercise therapy is contraindicated (including moderate or higher aortic aneurysm, severe hypertension, thrombophlebitis, embolism within 2 weeks, and serious other organ damage); congenital cardiovascular disease; renal failure with estimated glomerular filtration rate <30 mL/min/1.73m^2^; diagnosis of psychiatric disorders; malignant tumor; patients with HFA-PEFF score ≤4, New York Heart Association class I and IV; and patients under 65 or over 80 years of age. Of the 117 registrants, 18 were excluded from the analysis (six dropped out during the exercise intervention, seven lacked data on physical activity, two were hospitalized due to orthopedic disease, and three required an increase in medication).

**2.2 Calculated for sample size**

The sample size was calculated using the method described in a study by Edelmann et al. [14]. Forty-five samples were required for each group, calculated with a minimal clinically important difference of mean peakVO_2_ of 3.0 mL/min/kg, a standard deviation of 5.0 mL/min/kg, a significance level of 0.05, and a detection power of 0.9. We predicted a small number of dropouts in both the intervention and control groups and recruited 55 participants for each group.

**2.3 Diagnosis of heart failure with preserved ejection fraction (HFpEF)**

HFpEF was defined as the presence of clinical symptoms of heart failure (HF) (clinical HF symptoms were defined as subjective symptoms such as shortness of breath during exertion, orthopnea, and paroxysmal nocturnal dyspnea), a left ventricular ejection fraction >50%, and the presence of left ventricular diastolic dysfunction as defined by the American Society of Echocardiography/European Association of Cardiovascular Imaging (i.e., mean E/e′ >14, septal e′ <7 cm/s or lateral e′ <10 cm/s, tricuspid regurgitant velocity >2.8 m/s, left atrial volume index >34 mL/m^2^, where the above 3 points are considered satisfactory) [12]. Additionally, we calculated the HFA-PEFF score to diagnose HFpEF more accurately [13]. The definitions adopted for the HFA-PEFF criteria were based on the HFA-PEFF diagnostic algorithm for HFpEF in a consensus report proposed in 2019 by the Heart Failure Association of the European Society of Cardiology [13]. The HFA-PEFF score includes echocardiographic screening for functional or morphological abnormalities and the measurement of brain natriuretic peptide levels. In brief, 2 points are awarded if each major criterion is met, and 1 point if the sub-criteria are met. If the total HFA-PEFF score is <1 point, a diagnosis of HFpEF is unlikely, and investigation of alternative causes of the disease is warranted. An immediate diagnosis of HFpEF is recommended if the score is ≥5 points.

**2.4 Exercise training program**

All sessions were supervised by a cardiologist and physical therapist with specialized training in exercise training for patients with cardiovascular disease. The aerobic exercises were monitored using an ergometer (EC-MD100; CATEYE Co., Ltd., Osaka, Japan). Aerobic exercise intensity was determined based on the heart rate (HR) at the anaerobic threshold (AT) measured through cardiopulmonary exercise testing (CPET) with a symptomatic limit intensity (watt) of 1 min before AT. We also paid attention not to exceed Borg scale 13 in subjective fatigue during AT-intensity aerobic exercise. Since AT intensity does not excessively increase blood pressure or heart rate, it is possible to exercise without putting a heavy strain on the heart and vascular system. The karvonen method is famous for exercise prescriptions based on heart rate and can easily determine moderate exercise intensity (equivalent to 40–60% of peakVO_2_). It is also recommended to prescribe exercise within the range of 11 to 13 using the Borg scale, which is a subjective exercise intensity. However, caution is required in patients taking heart rate-suppressing drugs such as β-blockers and in elderly people with heart failure who do not exercise regularly. The reason for this is that the heart rate does not rise sufficiently, and the 40–60% of the maximum oxygen uptake, which is generally called moderate load, as well as the Borg scale may be overloaded. Resistance training was conducted using the following method in accordance with the guidelines for resistance training in patients with heart disease at the American College of Sports Medicine [15]. Using an elastic band (THERABAND; SAKAImed Co., Ltd., Tokyo, Japan), the training intensity was the rate of perceived exertion on a scale of 6 to 20 points. The chest and shoulders were indexed by subjective fatigue of approximately 11 to 13, 10 times for the upper arm, back, buttocks, and legs (two sets with a frequency of three times a week).

**2.5 Measurements of anthropometric parameters, biochemical data, and blood pressure**

Body height was measured to the nearest 0.1 cm using a wall-mounted stadiometer (DC250; Tanita Co. Ltd., Tokyo, Japan). The body weight of barefoot patients was measured to the nearest 0.1 kg using calibrated electronic digital scales (DC250; Tanita).

Brain natriuretic peptide, triglyceride, total cholesterol, high-density lipoprotein cholesterol, fasting plasma glucose, and fasting plasma insulin levels were measured using an enzymatic method. Hemoglobin A1c levels were determined using high-performance liquid chromatography. It was expressed in the National Glycohemoglobin Standardization Program unit. An enzyme solution (Qualijent TG and Choletest N HDL; Sekisui Medical Co., Ltd., Tokyo, Japan) was added to 2–3 μL of the sample. The mixture was heated at 37 °C for 5 min, and the absorbance was measured to determine its concentration. The absorbance of the enzyme solution was measured at two wavelengths (600 and 700 nm) using an automatic analyzer (LABOSPECT008; Hitachi High Technologies Co., Ltd., Tokyo, Japan).

Hypertension, diabetes mellitus, and dyslipidemia were determined according to the Japanese Diagnosis Criteria as follows: (1) systolic blood pressure (SBP) ≥130 mmHg and/or diastolic blood pressure ≥85 mmHg; (2) fasting plasma glucose level ≥126 mg/dL, 2-h value for the 75 g oral glucose tolerance test ≥200 mg/dL, casual plasma glucose level ≥200 mg/dL, hemoglobin A1c level ≥6.5%; (3) triglyceride levels ≥150 mg/dL; (4) and high-density lipoprotein cholesterol levels <40 mg/dL [21].

**2.6 Evaluation of pulmonary artery systolic pressure and mitral regurgitation**

The pulmonary artery systolic pressure was estimated from the peak flow velocity of tricuspid regurgitation, and the estimated right atrial pressure using the simplified Bernoulli equation. Right atrial pressure was estimated from the inferior vena cava diameter and respiratory variation. The normal right atrial pressure was set at 3 mmHg when the diameter of the inferior vena cava was <21 mm, and sniffing reduced the diameter by >50%. In particular, a pressure of 15 mmHg was set for inferior vena cava diameters >21 mm with <50% sniff variation or <20% resting respiratory variation. If none of these conditions were met, the middle 8 mmHg was used as the estimated right atrial pressure [24].

For mitral regurgitation (MR), a visual-qualitative evaluation was performed first. Tracing was performed at the time when the regurgitant jet could be visualized to its maximum. A small, thin central jet was considered mild MR, a jet covering >50% of the LA area was considered severe MR, and an intermediate jet area between mild and severe was considered moderate MR. For quantitative evaluation, MR was recorded by continuous wave Doppler, the maximum waveform was traced, and the effective regurgitant orifice area (EROA) and mitral regurgitation volume (MR volume) were automatically measured by the proximal isovelocity surface area method. The European Association of Cardiovascular Imaging strongly recommends the use of the proximal isovelocity surface area method to quantify the severity of [25]. The severity was graded as “mild” (EROA <0.20 cm^2^ or <MR volume 30 mL), “moderate” (EROA 0.20–0.39 cm^2^ or MR volume 30–59 mL), or “severe” (EROA ≥0.40 cm^2^ or MR volume ≥60 mL) [26]. In our study, the presence of severe valvular disease requiring surgery was one of the exclusion criteria. All MR severity data presented in Table 2 are based on the quantitative evaluation.

**2.7 Speckle-tracking imaging**

After manually tracing the endocardial border and selecting the appropriate wall thickness, the software automatically identified six segments in each view and tracked the motion of the acoustic markers. For systolic function assessment, parameters assessed from the myocardial deformation curves, including peak strain (defined as the most significant negative value on the strain curve) and peak systolic and early diastolic strain rates, were averaged from all measured segments.

**2.8 Measurement of the epicardial adipose tissue thickness**

Two-dimensional guided M-mode echocardiography was performed using a 2.5-MHz transducer with the participants in the left lateral decubitus position. Parasternal long- and short-axis views were measured from the trailing edge to the leading edge of the end-systolic right ventricular free wall. This information was saved for offline analysis. Then, the mean values measured in the parasternal long- and short-axis views were calculated.

**2.9 Measurement of exercise capacity and hemodynamic response**

The peakVO_2_ and AT were determined using the ramp loading method with an increase of 10 W/min after a rest period of 4 min and a warm-up at 0 W for 4 min. The highest oxygen uptake after more than 30 s of exercise was peakVO_2_.

The peakVO_2_ was defined as the highest VO_2_ recorded during exercise [31]. The AT was determined using the V-slope method described by Beaver et al. [32].

A respiratory analyzer (AE300S; Minato Medical Science Co., Ltd., Tokyo, Japan) was used in breath-by-breath mode to measure expiratory gas. Exhaled gas data were analyzed using an average of 10 respiratory cycles.

The number of pedal revolutions during bicycle movement was 60 revolutions/min (rpm). Heart rate was constantly monitored at rest, during exercise, and during recovery using an electrocardiogram monitor (ML4500; Fukudadenshi Co., Ltd. Tokyo, Japan). The endpoints of the bicycle movement were as follows: (1) leveling off of VO_2_, (2) decrease in SBP by 10 mmHg with an exercise load and SBP of 250 mmHg or more, (3) rate of perceived respiratory exertion and rate of perceived lower-extremity exertion >17, (4) a respiratory exchange ratio of >1.15, and (5) a pedal speed of <50 rpm (≥3 s). This was considered complete if at least one of the five conditions was satisfied.

Chronotropic incompetence was determined to be <80% of the predicted maximum HR, according to Laforgia et al. [33]. Based on a report by Cole et al., an abnormal HRR value was defined as a reduction of 12 beats/min or less from the heart rate at peak exercise [34].

**2.10 Measurement of physical activity and nutrition intake**

To objectively assess physical activity, movement-related calorie consumption during physical activity and the number of steps taken were measured continuously for one week using a Lifecorder (Suzuken Co., Ltd., Nagoya, Japan) worn in the lumbar region by the study participants. Data were accepted when ≥75% of the Lifecorder was attached per unit of time and rejected when ≥25% of defects were confirmed due to non-attachment. The defect criterion was continuous non-wearing for ≥3 h out of 12 h (body movement level 0 was regarded as non-wearing).

The dietary surveys used were the food intake frequency survey (FFQg version 5.0; Kenpakusya Co., Ltd., Tokyo, Japan) and Excel Nutrition (Eiyoukun version 8.0; Kenpakusya Co., Ltd., Tokyo, Japan). This method is a survey developed based on domestic data, and its validity has been suggested in comparison with dietary recording methods [36]. A survey was conducted on all subjects, and the total energy intake was kcal, and the intake ratios of carbohydrate (carbohydrate intake), fat intake, and protein intake to total energy intake were expressed in percentage (% carbohydrate intake, % fat intake, and % protein intake, respectively).

**2.11 Diagnosis of sarcopenia**

The skeletal muscle index was calculated by dividing the limb skeletal muscle mass measured using bioelectrical impedance analysis (InBody720, BIOSPACE Co., Urbandale, IA, USA) by the square of height (m).

Grip strength was measured in the dominant arm using the Smedley-type grip strength (T.K.K.5401, Takei Scientific Instruments Co., Tokyo, Japan).

The five-time chair stand test starts with a chair 40 cm in height. The time required to repeat the motion five times as fast as possible was measured in 0.01-s units.
